# 2,4-D resistance in wild radish: reduced herbicide translocation via inhibition of cellular transport

**DOI:** 10.1093/jxb/erw120

**Published:** 2016-03-19

**Authors:** Danica E. Goggin, Gregory R. Cawthray, Stephen B. Powles

**Affiliations:** ^1^Australian Herbicide Resistance Initiative, University of Western Australia, 35 Stirling Highway, Crawley 6009, Australia; ^2^School of Plant Biology, University of Western Australia, 35 Stirling Highway, Crawley 6009, Australia

**Keywords:** ABCB transporters, auxin mimics, Brassicaceae, herbicide resistance, translocation, weeds.

## Abstract

Resistance to auxinic herbicides is increasing in a range of dicotyledonous weed species, but in most cases the biochemical mechanism of resistance is unknown. Using ^14^C-labelled herbicide, the mechanism of resistance to 2,4-dichlorophenoxyacetic acid (2,4-D) in two wild radish (*Raphanus raphanistrum* L.) populations was identified as an inability to translocate 2,4-D out of the treated leaf. Although 2,4-D was metabolized in wild radish, and in a different manner to the well-characterized crop species wheat and bean, there was no difference in metabolism between the susceptible and resistant populations. Reduced translocation of 2,4-D in the latter was also not due to sequestration of the herbicide, or to reduced uptake by the leaf epidermis or mesophyll cells. Application of auxin efflux or ABCB transporter inhibitors to 2,4-D-susceptible plants caused a mimicking of the reduced-translocation resistance phenotype, suggesting that 2,4-D resistance in the populations under investigation could be due to an alteration in the activity of a plasma membrane ABCB-type auxin transporter responsible for facilitating long-distance transport of 2,4-D.

## Highlight

Reduced translocation of 2,4-D confers resistance in wild radish, and is due to inhibition of phloem loading, rather than enhanced metabolism or sequestration of the herbicide.

## Introduction

Herbicides that mimic the key plant hormone auxin (indole-3-acetic acid; IAA) have been in use for almost 70 years, and it has taken nearly as long for their mode of action to be identified. As reviewed comprehensively in Grossmann (2010), synthetic auxins, which are much less prone to degradation or inactivation than IAA, bind to the soluble auxin receptor TIR1 and/or to one of its several analogues, causing increased expression of auxin-responsive genes, particularly those responsible for synthesis of ethylene and abscisic acid (ABA). The treated plant suffers from a continuously high level of auxin signalling and displays the classical symptoms of early uncontrolled tissue growth and epinasty, followed by growth inhibition and death (Grossmann, 2010). Recent studies have shown that epinasty, long thought to be caused by the high levels of ethylene, is actually due to oxidation of the actin cytoskeleton by the hydroxyl radicals generated by up-regulated xanthine oxidase activity (Pazmiño *et al.*, 2013; Rodríguez-Serrano *et al.*, 2014). Ethylene contributes to the generation of reactive oxygen species (Rodríguez-Serrano *et al.*, 2014), as does the inhibition of photosynthesis caused by ABA-mediated stomatal closure (Grossmann, 2010). In general, grass species are naturally resistant to auxinic herbicides, apparently through a combination of altered perception, metabolic detoxification. and the anatomy of their vascular systems (reviewed in Robertson and Kirkwood, 1970' Grossmann, 2010). At least 31 dicotyledonous weed species, under selection with synthetic auxin herbicides, have evolved resistance (Heap, 2016). In view of the complicated mode of action of auxinic herbicides, the evolution of resistance in weeds is generally treated as a non-target-site-based phenomenon.

Mechanisms of non-target-site-based herbicide resistance include reduced herbicide uptake, where the leaf cuticle or other structural barrier prevents absorption of the herbicide into the mesophyll (e.g. Kohler *et al.*, 2004); reduced translocation, where a normally mobile herbicide remains at its site of application rather than moving systemically through the plant in the phloem and/or xylem stream (e.g. Riar *et al.*, 2011); and detoxification, where the herbicide is metabolized to a harmless form and/or conjugated to large, polar molecules such as sugars or glutathione (reviewed in Yuan *et al.*, 2007). Often, reduced herbicide translocation is due to sequestration of the herbicide or its conjugates in the vacuole or the apoplast, away from both the vascular system and the site of herbicide action (Yuan *et al.*, 2007; Ge *et al.*, 2010).

In spite of this general knowledge, there is a lack of definitive molecular or biochemical information about the mechanisms conferring evolved resistance to auxinic herbicides in weeds. Potential detoxification of auxin mimics was reported in resistant populations of *Carduus nutans* (Asteraceae) (Harrington and Woolley, 2006), *Kochia scoparia* (Amaranthaceae) (Keith *et al.*, 2011), and *Stellaria media* (Caryophyllaceae) (Coupland *et al.*, 1990), whereas increased expression of peptidylprolyl *cis*-*trans* isomerase appeared to lead to herbicide hydroxylation in *Synapsis arvensis* (Brassicaceae) resistant to 3,6-dichloro-2-methoxybenzoic acid (dicamba) (Yajima *et al.*, 2004). Reduced auxinic herbicide absorption and/or translocation conferred resistance in *Glechoma hederacea* (Lamiaceae) (Kohler *et al.*, 2004), *Lactuca serriola* (Asteraceae) (Riar *et al.*, 2011), *Galeopsis tetrahit* (Lamiaceae) (Weinberg *et al.*, 2006). and *Centauria solstitialis* (Asteraceae) (Fuerst *et al.*, 1996), but the molecular basis for this was not explored. A study on 2-methyl-4-chlorophenoxyacetic acid (MCPA) resistance in *Raphanus raphanistrum* L. (wild radish: Brassicaceae), in which MCPA translocation to the roots was higher in the resistant plants, suggested that the resistant plants may exude the herbicide from the roots, but this was not directly demonstrated (Jugulam *et al.*, 2013). Differential perception of auxinic herbicides at the plasma membrane and an alteration in the auxin signalling pathway were identified in resistant populations of *Brassica kaber* (Brassicaceae) (Mithila and Hall, 2005) and *Echinochloa crus-galli* (Poaceae) (Xu *et al.*, 2013), respectively. In *Sisymbrium orientale* (Brassicaceae), a single, so-far unidentified gene appears to be responsible for 2,4-dichlorophenoxyacetic acid (2,4-D) resistance (Preston and Malone, 2014).

Wild radish is a troublesome weed in many regions of the world, and is the most economically damaging dicotyledonous weed in the Western Australian grain belt, where it has evolved resistance to auxinic herbicides. In the first random survey of 2,4-D resistance in this region in 2004, 60% of wild radish populations contained 2,4-D-resistant individuals (Walsh *et al.*, 2007). By the latest survey in 2010, this had increased to 76%, and is likely to rise further due to continued reliance on 2,4-D in the face of even higher levels of resistance (84%) to the commonly used sulphonylurea herbicides (Owen *et al.*, 2015). The aim of the current study was to elucidate the currently unknown mechanism(s) of evolved 2,4-D resistance in two wild radish biotypes, and then to explore its biochemical basis, in order to develop strategies to ameliorate 2,4-D resistance in this weed species.

## Materials and methods

### Plant material

A 2,4-D-susceptible (S) population was originally collected from the northern grain belt of Western Australia near Yuna (28.33°S, 114.96°E) in 1999 (Walsh *et al.*, 2004). Seed stocks have been maintained over the years by allowing plants from this population to cross-pollinate amongst themselves (using an insect vector) while excluding pollen from other wild radish populations. The original field-evolved 2,4-D-resistant populations were collected from Wongan Hills (30.88°S, 116.51°E) in 2002 (population R1) and Eneabba (29.82°S, 115.27°E) in 2010 (population R2); these populations displayed survival of ~30% (Walsh *et al.*, 2009) and ~32% (Owen *et al.*, 2015), respectively, when sprayed with the recommended field rate (500g active ingredient ha^−1^) of formulated 2,4-D amine. All three biotypes displayed leaf curling, petiole elongation, and epinasty after spraying with 2,4-D, but resistant individuals were able to produce asymptomatic new growth within 7 d of treatment. In contrast, susceptible plants showed gradual worsening of symptoms, no new growth, and eventual death (Supplementary Fig. S1 at *JXB* online). To generate more homogeneous populations for the current study, the R1 and R2 populations were selected over two generations with 2,4-D. Fifty plants at the two- to three-leaf stage were sprayed with 500g ha^−1^ formulated 2,4-D amine (Amicide 625 or Amicide Advance 700) (Nufarm Australia) as described in Owen *et al.* (2015), and the 20–25 survivors with the greatest amount of new growth at 21 d after spraying were allowed to cross-pollinate within their respective populations. Seeds produced from the 25 best growing survivors of the second round of selection were used as the R germplasm for the current study.

Seeds of S and R were sown in potting mix (50% composted pine bark, 25% washed river sand, 25% peat moss) in individual 250ml polystyrene cups with drainage holes, and seedlings were grown under controlled conditions in a growth cabinet (20/15 °C day/night; 12h photoperiod of cool white fluorescent light at 90 μmol m^−2^ s^−1^). Plants were watered regularly and fertilized with commercial soluble fertilizer once a week. Visual inspection of seedlings grown under controlled conditions showed that individuals from the twice-selected R1 population often had smaller, more deeply divided leaves than S seedlings and had produced four or five of these small leaves in the time that the S seedlings had produced two large leaves. The R2 seedlings more closely resembled S seedlings, except that the leaves were slightly thicker and larger and held on shorter, reddish-coloured petioles. Examples of S, R1, and R2 seedlings are shown in Supplementary Fig. S1. As the plants grew older, the differences between them were less apparent, although R1 plants remained more slender and produced fewer, smaller seeds (not shown).

For some experiments, plants were transferred to a hydroponic system before 2,4-D and other treatments were imposed. In these cases, seedlings at the two-leaf stage were removed from the cups, the potting mix gently washed from the roots, and the seedlings placed in individual 85ml glass tubes containing half-strength Arabidopsis inorganic nutrient solution (Okada *et al.*, 1991). Seedlings were supported by foam floats so that the roots were submerged and the entire shoot was above the rim of the tube and exposed to the air. The tubes were wrapped in foil to minimize algal growth in the nutrient solution. The solution was not constantly aerated, but was changed daily. Plants were given a recovery time of 24h after transferral to hydroponics, before commencement of treatments.

### Experimental design

The following possible mechanisms of 2,4-D resistance were tested, using the methods detailed below: (i) inability of 2,4-D to penetrate the leaf cuticle; (ii) reduced uptake at a cellular level; (iii) reduced translocation out of the treated leaf; (iv) enhanced metabolism of parent 2,4-D to less toxic compounds; (v) sequestration of 2,4-D and/or its metabolites in the vacuole, apoplast, or endoplasmic reticulum; (vi) reduced sensitivity to auxins at the cellular level; and (vii) enhanced antioxidant capacity of treated tissue.

### Leaf uptake and translocation of [^14^C]2,4-D

Unless otherwise specified, all chemicals were from Sigma-Aldrich. First or second (attached) leaves of seedlings at the three-leaf stage were treated (one leaf per plant, 3–6 plants per biotype for each experiment) with ten 0.5 μl droplets of [^14^C]2,4-D acid (ring ^14^C [U]; specific activity 2.035 GBq mmol^−1^) (American Radiolabeled Chemicals) diluted in 0.1% (v/v) Tween-20, such that a total of 3 kBq was applied per leaf, avoiding the mid-vein. Plants were incubated in the growth cabinet for 24h (unless otherwise specified) and treated leaves were then rinsed with 10ml of 50% (v/v) methanol containing 0.1% (v/v) Tween-20. Plants were gently uprooted from the potting mix, the roots rinsed in 10ml of methanol/Tween-20, and the intact plants were then pressed and dried at 70 °C for 48h. The leaf and root washes were assayed for ^14^C by liquid scintillation counting (LSC) using a Packard Tri-Carb 1500 Liquid Scintillation Analyzer, whereas pressed plants were exposed to a BAS-IP MS2040 storage phosphor screen (GE Healthcare) for 24h. Plates were read on a Personal Molecular Imager (BioRad).

To quantify the extent of 2,4-D translocation, the pressed plants were then separated into (i) treated leaf plus petiole; (ii) untreated leaves plus petioles; (iii) stem (including the apex and the smallest unexpanded leaf); and (iv) roots. The amount of 2,4-D in each plant part was expressed as the percentage of total ^14^C recovered (Bq g^−1^ DW) from the whole plant. Plant parts were pulverized and extracted in 100% methanol at room temperature for 3 d, followed by digestion of the insoluble material with 0.5M KOH in 50% (v/v) methanol (Hamburg *et al.*, 2001) at 35 °C for 5 d. The combined extract was measured by LSC. Recovery of applied ^14^C from fresh plant parts was always >80%, but, when dried plants were used, recovery decreased to 70%. However, the relative distribution of ^14^C in plant parts extracted when fresh or dry remained the same (data not shown).

In some experiments, the effect of a pre-treatment with known transport inhibitors was assessed. Plants were grown hydroponically, with 10 μM 1-naphthylphthalamic acid (NPA), 2,3,5-triiodobenzoic acid (TIBA), (*R*)-2-(3,4-dimethoxyphenyl)-5-{[2-(3,4-dimethoxyphenyl)ethyl]-(methyl)amino}-2-prop-2-ylpentanenitrile hydrochloride (verapamil), or 6-[(2S,4R,6E)-4-methyl-2-(methylamino)-3-oxo-6-octenoic acid]-7-l-valine-cyclosporin A (valspodar) included in the nutrient solution 8h before application of [^14^C]2,4-D to the leaf. Controls contained 0.1% DMSO, equivalent to the concentration of solvent added with the inhibitors. In a separate experiment, soil-grown S plants were treated by hand-spraying the treated leaf with 10 μM inhibitor solutions in 0.1% (v/v) Tween-20, 1h before [^14^C]2,4-D application. NPA and TIBA are inhibitors of auxin efflux with different modes of action (NPA binds to ABCB-type auxin transporters and their chaperone protein TWD1, whereas TIBA inhibits cycling of PIN-type auxin transporters between the plasma membrane and endosomes; reviewed in Zhu and Geisler, 2015), and verapamil and valspodar inhibit ABCB-type membrane transporters in general (Shukla *et al.*, 2011).

Pilot studies in which plants were sprayed with 0.25× the recommended rate of formulated 2,4-D amine (i.e. 125g ha^−1^) 1h before [^14^C]2,4-D application showed that the 2,4-D pre-treatment had no effect on [^14^C]2,4-D uptake and translocation in either the S or R biotypes (data not shown). Therefore, translocation experiments were routinely performed without a 2,4-D pre-treatment, because the auxin overdose symptoms in pre-treated plants (stem and petiole swelling and brittleness, tight leaf curling) made subsequent handling of the plants more difficult.

### Localization of 2,4-D in treated leaves

Isolation of apoplastic fluid from [^14^C]2,4-D-treated wild radish leaves was based on the vacuum infiltration–centrifugation method of Rohringer *et al.* (1983), although it was necessary to pressurize leaf pieces in a syringe to achieve full infiltration (Witzel *et al.*, 2011). The apoplastic fluid (diluted in the infiltration buffer) was assayed for NADH-malate dehydrogenase (MDH) activity (Bergmeyer and Bernt, 1974) as a marker of contamination by the symplast, and for ^14^C by LSC. The leaf pieces were then crushed and centrifuged at 12 000 *g* for 10min at 4 °C, and the resulting liquid (symplast) was also assayed for MDH and ^14^C activity. Microsomes were isolated from [^14^C]2,4-D-treated leaves using the MgCl_2_ precipitation method of Diesperger *et al.* (1974). MDH activity was used as a marker for the cytosol/mitochondria/chloroplasts, and NADPH-cytochrome *c* reductase (assayed according to Hodges and Leonard, 1974) as a marker for the endoplasmic reticulum. Total protein was measured according to Bradford (1976) using BSA as a standard. For both experiments, three replicates each consisting of three [^14^C]2,4-D-treated leaves from different individuals were extracted for each biotype.

### Leaf disc [^14^C]2,4-D influx/efflux experiments

Plants at the five- to six-leaf stage were sprayed with 125g ha^−1^ formulated 2,4-D amine, 18–24h prior to experiments. Young, expanded leaves were then excised and washed, their lower epidermis was abraded with fine sandpaper, and they were submerged in water, so that discs of 10mm diameter could be punched out with a cork borer. Discs (six replicates, three discs per replicate, cut from three plants) were floated on pre-incubation medium [20mM MES (pH 5), 250mM mannitol, 0.5mM CaCl_2_, 0.25mM MgCl_2_, and 10mM sucrose] for 30min at 22 °C with gentle agitation (Delétage-Grandon *et al.*, 2001) and then transferred to individual wells for incubation with [^14^C]2,4-D in 150 μl of influx medium in a humid environment to minimize evaporation. Influx medium was the same as the pre-incubation medium with the addition of 63 Bq (per well) of [^14^C]2,4-D and 0.1mM unlabelled 2,4-D. Pilot studies showed that unlabelled 2,4-D concentrations up to 2.5mM did not affect the rate of influx (data not shown). Following incubation in influx medium for 2–180min, discs were washed three times for 2min each in washing medium (influx medium lacking [^14^C]2,4-D) and then extracted by steeping in 500 μl of 0.5M KOH in 50% (v/v) methanol (the three discs for each replicate were extracted together) for several hours, followed by crushing with a steel rod. The clarified supernatant was assayed by LSC to measure the amount of ^14^C taken up by the discs.

Efflux of 2,4-D was monitored by pre-loading discs with [^14^C]2,4-D for 3h as described for the influx experiment above. Another set of discs (pooled biotypes, three replicates) was also loaded with Neutral Red (a stain accumulating in the vacuole; Burla *et al.*, 2013), applied as a 0.01% (w/v) solution in the influx buffer, to measure its efflux over 24h. After washing, discs were transferred to fresh efflux medium of the same composition as the washing medium (1ml per set of three discs), and the efflux buffer was sampled and replaced over a time course of 2min to 24h. Discs were then extracted as above, and the ^14^C in the loading, washing, and efflux buffers was also counted. All steps were performed at 22 °C. A duplicate experiment was set up in which 16 [^14^C]2,4-D-loaded discs from each biotype were collected after 16h incubation in efflux medium and extracted in 100% methanol, for analysis by TLC (see below).

The amount of ^14^C taken up as measured directly in discs harvested immediately after the influx phase was very similar to the amount deduced from the sum of ^14^C in the efflux medium and in the discs harvested after the efflux period (Supplementary Table S1). Therefore, in the efflux experiments, the amount of ^14^C loaded into the discs was deduced in order to minimize sample processing. The mean recovery of applied ^14^C from all incubation buffers, washes, and extracts was 99±2, 97±2, and 97±2% for the S, R1, and R2 biotypes, respectively.

### Analysis of 2,4-D metabolism

For assessment of 2,4-D metabolism in shoot tissue, plants (1–3 plants per experiment, three or four independent experiments) were sprayed with 125g ha^−1^ formulated 2,4-D amine 1h before 3 kBq of [^14^C]2,4-D was applied to a single attached leaf as described above. In some experiments, a formulation of the cytochrome P450 mono-oxygenase (P450) inhibitor malathion (David Gray’s Malathion Garden Spray: David Gray & Co., Perth, Australia) was sprayed at a rate of 1000g ha^−1^ on the plants 30min before the application of formulated 2,4-D amine. Metabolism of 2,4-D in root tissue was investigated by placing the roots of twelve 6-day-old seedlings [germinated on 0.6% (w/v) agar] into 2.5ml of half-strength nutrient solution containing 2 kBq of [^14^C]2,4-D and 10 μM unlabelled 2,4-D. After 96h (unless otherwise indicated) incubation with [^14^C]2,4-D under controlled conditions, tissues were harvested. Based on the different translocation pattern of leaf-applied [^14^C]2,4-D in the S and R plants (see the Results), whole S shoots but only treated R leaves were harvested for analysis, in order to maximize ^14^C recovery. Before processing, treated leaves were washed in methanol/Tween-20 as described above, whereas treated roots were washed in nutrient solution containing 10 μM unlabelled 2,4-D. Recovery of applied ^14^C from washes plus extracts was 80–100%.There was no post-harvest, non-enzymatic degradation of 2,4-D, as shown by analysis of untreated tissues that were extracted in parallel with treated plants, with 3 kBq of [^14^C]2,4-D added during the extraction step (data not shown). As positive controls for efficient 2,4-D metabolism, a resistant cereal (wheat: *Triticum aestivum* cv. Bonnie Rock) and a susceptible dicot (bean: *Phaseolus vulgaris* cv. Brown Beauty) were included in some experiments.

Harvested shoot and root tissue was extracted in cold 100% methanol according to López-Villalobos *et al.* (2004). The extracts were evaporated to dryness under a stream of air and then resuspended in a small volume of 100% methanol (for TLC), 50% (v/v) methanol in water (for HPLC). or acidified water (pH ~2, for partitioning). The latter samples were partitioned three times against diethyl ether and then 1-butanol according to Chkanikov *et al.* (1976). Following butanol partitioning of the first aqueous phase, none of the ^14^C-labelled compounds was detectable in the remaining water phase, as checked by LSC. Mild chemical hydrolysis by 1M NaOH (room temperature) or 2M HCl (95 °C) followed by re-partitioning was performed on partitioned extracts according to Hamburg *et al.* (2001). Separate enzymatic hydrolysis of the ether and butanol fractions by β-glucosidase was carried out as described in Laurent *et al.* (2000), except that an incubation time of 4h (at 30 °C) was used.

For TLC analysis of metabolites, known amounts (80–670 Bq) of ^14^C-containing sample dissolved in methanol were spotted onto 10×10cm silica gel TLC plates (Sigma-Aldrich), alongside authentic [^14^C]2,4-D. Plates were developed in toluene:2-butanone:acetic acid (45:55:3, v/v/v) according to López-Villalobos *et al.* (2004). Phosphorimage signal intensity was quantified by the Quantity One program (Bio-Rad; version 4.2.1), using a standard curve of 80–330 Bq of [^14^C]2,4-D. Standard [^14^C]2,4-D migrated at an *R*_f_ of 0.53.

HPLC analysis was performed using a modified version of protocol 2 from Hamburg *et al.* (2001). A 3.9×150mm Nova-Pak C_18_, 4 μm particle (Waters) column was used, with a mobile phase flow rate of 1.5ml min^−1^ at 23 °C. The mobile phase (acidified with 1% formic acid) was a linear gradient of 15–30% acetonitrile in water over 20min, followed by a second linear gradient of 30–100% acetonitrile over 5min, then held at 100% acetonitrile for 10min followed by re-equilibration with 15% acetonitrile for 15min. A β-RAM detector (IN/US Systems Inc, Pine Brook, NJ, USA) was used to monitor ^14^C eluting from the column. Under these conditions, standard [^14^C]2,4-D eluted at 24.3min.

### Auxin response in roots and shoots

To assess the physiological response of the S, R1, and R2 wild radish biotypes to 2,4-D and other auxins, root elongation (which is inhibited by high concentrations of auxin) and pro- and antioxidant levels were measured in young seedlings. Seeds were surface-sterilized in a 1:5 dilution of commercial bleach containing 0.1% (v/v) Tween-20, sown on 0.6% (w/v) agar and germinated in the dark for 3 d at room temperature. The length of the primary root of 3-day-old seedlings was recorded, and the seedlings transferred to agar containing the appropriate auxin treatment (see below) and placed under controlled conditions (20/15 °C, 12h photoperiod). After a further 7 d, the length of the primary root was again recorded. Treatment agar contained 2,4-D, MCPA, 2-(4-chloro-2-methylphenoxy)propanoic acid (mecoprop), dicamba, IAA, or 1-naphthylacetic acid (NAA) at concentrations of 0, 0.01, 0.1, 0.5, and 1 μM, with additional treatments of 10 μM and 50 μM included for 2,4-D and dicamba. Each treatment consisted of three replicates of five seedlings each. Controls contained 0.1% (v/v) ethanol, equivalent to the amount added with the auxin treatments. To check for 2,4-D localization in agar-grown seedlings, six seedlings of each biotype were placed on agar containing 0.5 μM unlabelled 2,4-D plus 6 kBq of [^14^C]2,4-D for 7 d, then washed in 0.5 μM unlabelled 2,4-D, and pressed, dried, and phosphorimaged as described above.

Ascorbic acid, glutathione, and H_2_O_2_ were measured in seedlings (three replicates of five seedlings each) that had been incubated on agar containing 0.1 μM 2,4-D for 3 d, alongside untreated controls. This 2,4-D concentration was selected because it resulted in almost complete inhibition of root elongation in the S biotype, but had little effect on the R biotypes. For measurement of pro- and antioxidants in young, expanded leaves, plants were grown in potting mix and leaves were sprayed with 500g ha^−1^ formulated 2,4-D amine. After 24h, the treated leaves were rinsed with water, harvested, and immediately snap-frozen in liquid nitrogen before analysis of the relevant compounds. An incubation time of 24h was selected because leaf curling and epinasty symptoms were pronounced, but the leaves had not yet started to desiccate. The treated and untreated leaves of S plants were also assayed for ascorbate and glutathione at 7 d after spraying. Each treatment consisted of three (H_2_O_2_) or six (ascorbate/glutathione) replicates of 2–3 leaves from different individuals.

Tissue H_2_O_2_ was measured colorimetrically using the ferrous sulphate and xylenol orange method (Minibayeva *et al.*, 2009). The validation method of Cheeseman (2006) was used to confirm that extracts with a tissue:buffer ratio of 1:100 or 1:200 did not interfere with the colour reaction (data not shown). Recovery of H_2_O_2_ added to extractions was 105±3%. Leaf ascorbate and glutathione were quantified spectrophotometrically in leaf tissue extracted in 5% (w/v) sulphosalicylic acid (Goggin *et al.*, 2011), and the half-cell reduction state of glutathione (E_GSSG/2GSH_) was calculated according to Schafer and Buettner (2001). Recovery of ascorbate and glutathione added to extractions was 97±6% in both cases.

### Statistical analysis

Data were analysed using one- and two-factor ANOVA and the least significant difference test at a significance level of 5%. Dose–response curves were constructed and analysed using the ‘drc’ package in R (version 3.0.1) (R Core Team, 2013).

## Results

### Leaf uptake and translocation of [^14^C]2,4-D

High levels of [^14^C]2,4-D uptake occurred in all three biotypes, although uptake by R2 was slightly lower than in S and R1. Recovery of applied ^14^C from leaf surface washes at 24h after [^14^C]2,4-D application was 1.6±0.4, 2.2±0.5, and 4.3±0.9% in biotypes S, R1, and R2, respectively. Dipping the treated leaves briefly in diethyl ether, which dissolves the waxy cuticle and other components of the leaf surface but does not disrupt the cells (Hagin *et al.*, 1970), resulted in an increase of ^14^C recovered from the leaf surface to 14±1, 12±1, and 12±1% in S, R1, and R2, respectively, indicating that >85% of applied ^14^C had penetrated the epidermal tissue in all biotypes.

**Fig. 1.**
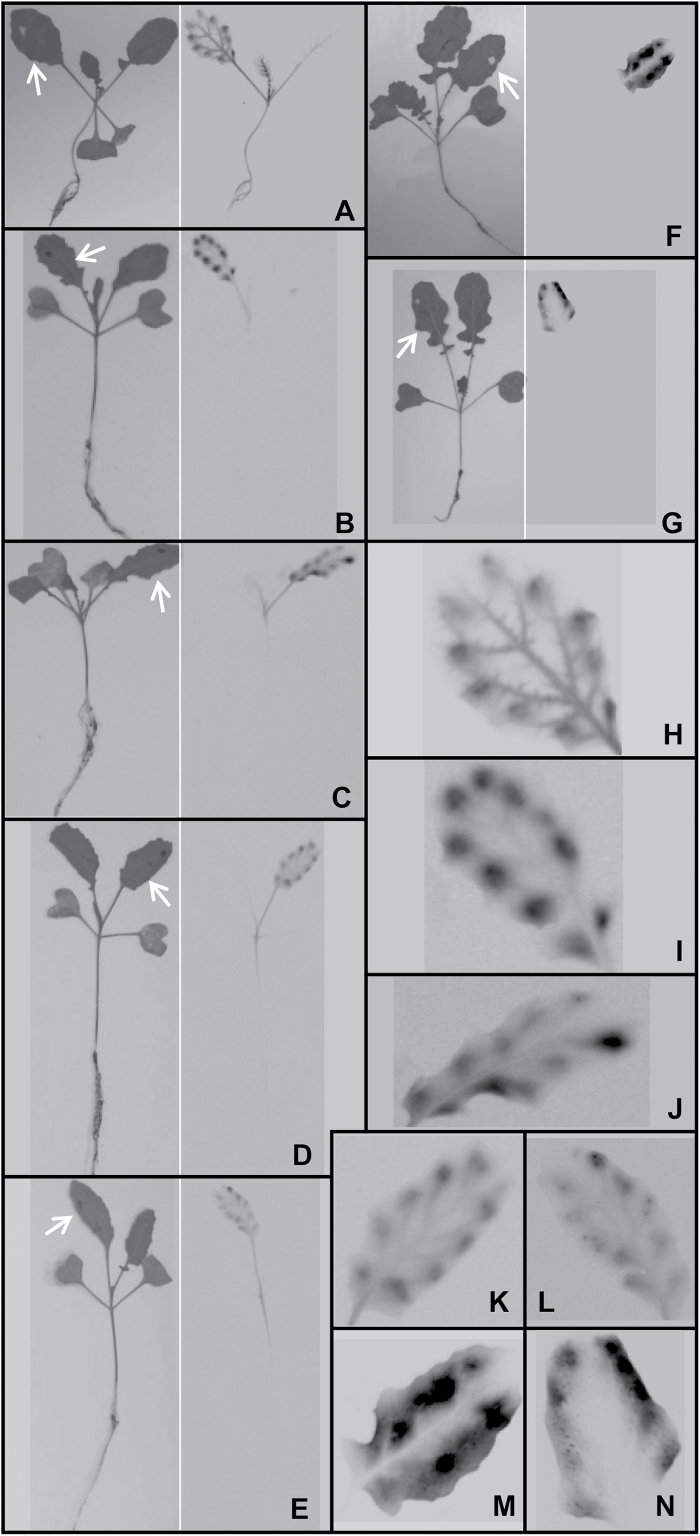
Visual representation of [^14^C]2,4-D translocation in susceptible (A–E), resistant R1 (F), and resistant R2 (G) wild radish seedlings. Photographs of each plant are shown to the left in each panel, and the corresponding phosphorimage of ^14^C localization to the right. Hydroponically grown seedlings were spotted with [^14^C]2,4-D on a single leaf (indicated with an arrow on the photograph) 8h after addition of 10 μM NPA (B), TIBA (C), verapamil (D), valspodar (E), or a solvent control (A, F, G) to the nutrient solution, and harvested after a further 24h. Representative plants from three independent replicates are shown, with close-ups of the treated leaves from A–G shown in H–N.

**Fig. 2.**
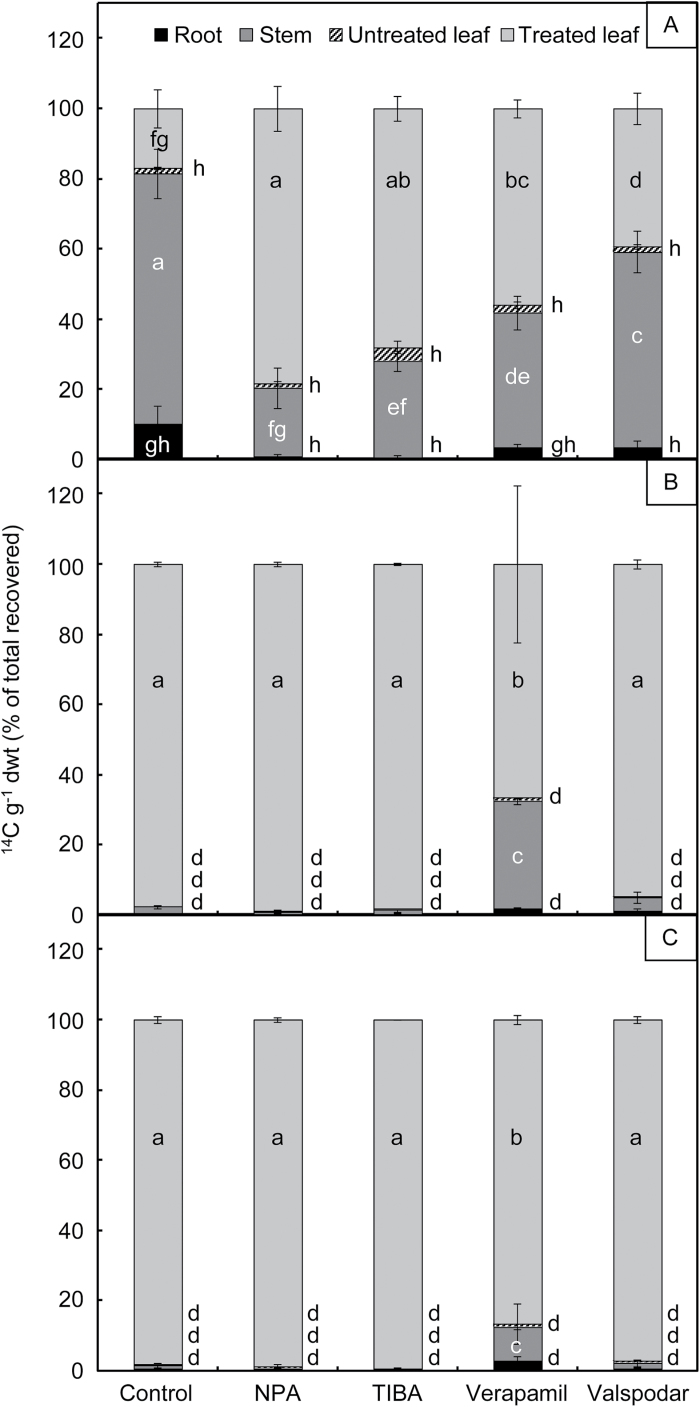
Quantification of [^14^C]2,4-D translocation in wild radish seedlings. The susceptible (A), resistant R1 (B), and resistant R2 (C) plants treated as described in the legend to Fig. 1 were separated into root, stem, untreated leaves, and treated leaf, and the ^14^C in digests of each plant part quantified by liquid scintillation counting. Data are expressed as the percentage in each plant part of the total Bq g^−1^ DW recovered, and are the mean ±SE of three independent replicates. Within each graph, different letters denote significant (*P*<0.05) differences between treatments and tissue types.

The extent of [^14^C]2,4-D translocation differed dramatically between the S and R biotypes. Phosphorimaging of the treated plants demonstrated that whilst ^14^C was distributed throughout the shoot of the S biotype after 24h (Fig. 1A), the label remained confined to the treated leaf in both R biotypes (Fig. 1F, G). The label had still not moved out of the treated leaves of R plants after 96h (data not shown). The difference in translocation between the S and R biotypes was confirmed and quantified by measuring ^14^C in the separate plant parts (Fig. 2). There was no detectable root excretion of ^14^C in either the S or R plants: LSC of the nutrient solution of hydroponically grown plants yielded a ^14^C recovery of <0.01%, 96h after the [^14^C]2,4-D was applied to the leaves.

When applied via the roots, the auxin efflux inhibitors NPA and TIBA significantly inhibited translocation of ^14^C out of the treated leaf in the S plants (Fig. 1B, C), with ≥4 times more ^14^C remaining in the treated leaf in the presence of the inhibitors (the amount of ^14^C in the stem was decreased by a similar factor) (Fig. 2A). The ABCB inhibitors verapamil and valspodar also inhibited movement of [^14^C]2,4-D from the treated leaf to the stem in the S biotype, but were not as effective as NPA and TIBA (Figs 1D, E, 2A). Close-ups of the treated leaves, showing the distribution of ^14^C between the vascular and mesophyll tissues, are shown in Fig. 1H–N. Foliar application of 10 μM inhibitors to the S plants did not influence translocation of [^14^C]2,4-D (data not shown), probably because the inhibitors did not efficiently penetrate the cuticle at such a low concentration. As expected, there was no effect of NPA or TIBA on the pattern of ^14^C distribution in the R plants (Fig. 2B, C; Supplementary Fig. S2). Valspodar also had no effect on the R biotypes, but verapamil caused an unexpected increase in translocation of [^14^C]2,4-D out of the treated leaf and into the stem (Fig. 2B, C; Supplementary Fig. S2).

### Localization of ^14^C in treated leaves

Isolation of apoplastic fluid and microsomes from [^14^C]2,4-D-treated leaves of the S and R populations indicated that only a very low percentage of recovered ^14^C was present in these fractions (Supplementary Table S2). Although there was a statistical difference in the amount of ^14^C recovered from microsomes from S (2.6%) compared with R (3.1–3.2%) plants (Supplementary Table S2), it is unlikely that this small numerical difference would contribute to 2,4-D resistance. In any case, the marker enzyme assays showed that the low level of contamination detected from the cytosol and organelles would largely account for the ^14^C present in the apoplast and microsomes (Supplementary Table S2).

Despite repeated attempts [using the method of Burla *et al.* (2013) and variations thereof], it was not possible to obtain high yields of pure vacuoles to use in [^14^C]2,4-D uptake experiments, and experiments in which vacuoles were isolated from [^14^C]2,4-D-treated leaves, and the distribution of ^14^C tracked throughout the procedure, also gave inconclusive results. The yield of ^14^C from these vacuole fractions was also too low to detect any potential 2,4-D metabolites inside the vacuoles. An attempt was also made to deduce the amount of ^14^C contained within the vacuoles by selectively lysing the plasma membrane and then the tonoplast membrane with increasing concentrations of DMSO (Delmer, 1979), but visual inspection of wild radish protoplasts following extreme DMSO treatment (20% DMSO for 2h at room temperature) showed that no lysis of either the plasma or tonoplast membranes had taken place (data not shown). As an alternative, [^14^C]2,4-D influx/efflux studies were performed on wild radish leaf discs.

### Leaf disc influx/efflux experiments

**Fig. 3.**
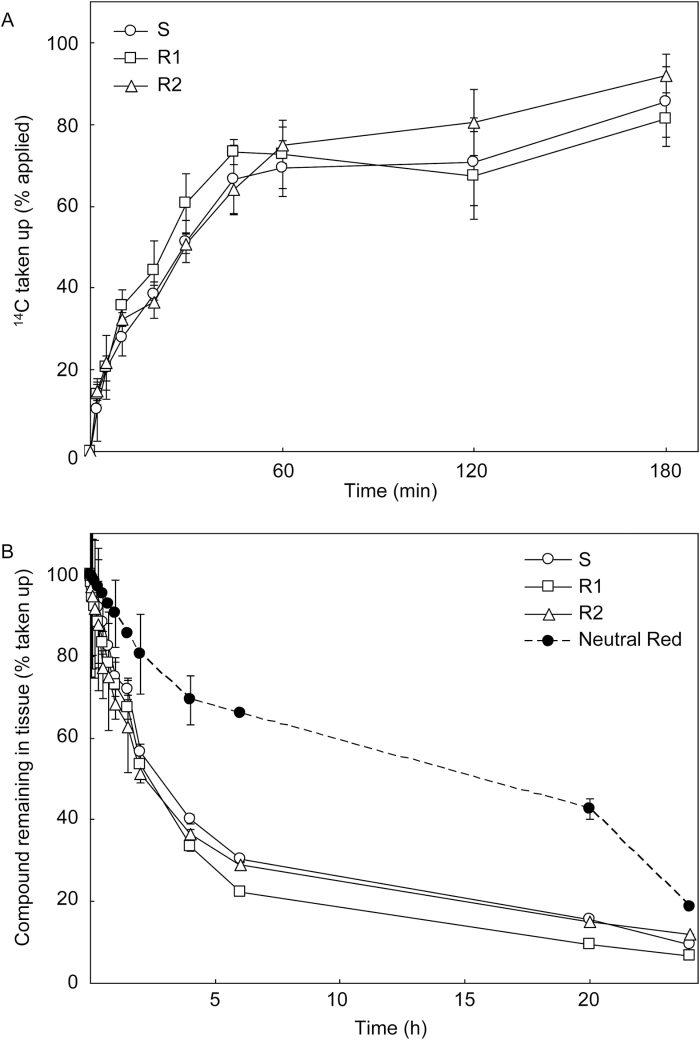
Time-course of [^14^C]2,4-D uptake and efflux in wild radish leaf discs from the susceptible (S) and resistant (R1 and R2) plants. (A) Abraded discs were floated on buffer containing [^14^C]2,4-D, and tissue ^14^C accumulation was measured over 2–180min. (B) Other discs were loaded with [^14^C]2,4-D or Neutral Red for 3h, then washed and transferred to unlabelled buffer to monitor ^14^C and Neutral Red efflux over 2min to 24h. The ^14^C remaining in the discs at the end of either the influx or efflux phase, as appropriate, was measured by liquid scintillation counting and expressed as a percentage of ^14^C applied (influx) or of the total ^14^C taken up by the discs (efflux). Values are means ±SE (*n*=6; three discs per replicate). The Neutral Red data were obtained from an experiment using discs pooled from all three biotypes (*n*=3; nine discs per replicate).

There was no significant difference between S and R biotypes in the rate or extent of [^14^C]2,4-D influx or efflux in leaf discs (Fig. 3). Influx of ^14^C reached close to its maximum value (~35% of applied ^14^C) by 60min (Fig. 3A). During the efflux phase, 65% of the accumulated ^14^C in the tissue was lost in the first 4h (Fig. 3B). Between 4h and 20h there was a further slow decline in disc ^14^C until almost 90% had been effluxed into the buffer (Fig. 3B). Inclusion of 3mM MgATP in the efflux buffer had no effect on the rate of efflux (data not shown). Neutral Red, which accumulates in the vacuoles of living plant cells, effluxed more slowly from the leaf discs than did ^14^C (only 30% lost after 4h and 60% after 20h), but showed a sharp decline between 20h and 24h (Fig. 3B), suggesting that the leaf tissue may have started to lose integrity near the end of the experiment. Therefore, the 24h time point was not included in the interpretation of the results. Analysis by TLC of the ^14^C remaining in [^14^C]2,4-D-loaded S, R1, and R2 leaf discs after 16h incubation in unlabelled efflux buffer showed that although there were very faint signals corresponding to the 2,4-D metabolites (see below), the most intense signal migrated identically to parent 2,4-D (Supplementary Fig. S3).

### Metabolism of [^14^C]2,4-D

**Fig. 4.**
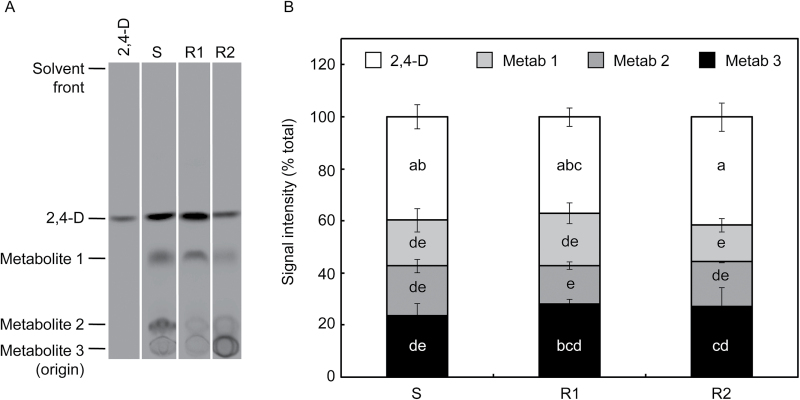
Metabolism of 2,4-D in wild radish plants. Extracts from [^14^C]2,4-D-treated plants were analysed by TLC, alongside a [^14^C]2,4-D standard. (A) Representative TLC separation of extracts from the susceptible (S) and resistant (R1 and R2) biotypes, harvested 96h after application of [^14^C]2,4-D to leaves of intact plants. (B) Quantification of ^14^C signals from parent [^14^C]2,4-D and its metabolites. Values are means ±SE of four independent experiments; different letters within bars denote significant differences (*P*<0.05) between means.

Quantification of the relative abundance of TLC bands of parent 2,4-D and its metabolites showed no difference in 2,4-D metabolic capacity between the S and R wild radish biotypes, with ~40% of the ^14^C signal on TLC plates being due to a compound migrating identically to parent 2,4-D (Fig. 4). To determine whether the R biotypes showed enhanced metabolism over a longer period or under greater herbicide stress, metabolites were also analysed in plants that had been incubated for 14 d following treatment, or that were exposed to a pre-treatment of unlabelled 2,4-D amine at double, rather than a quarter, the recommended rate. Neither of these treatments affected the metabolite profile in either the S or R biotypes, although the higher herbicide dose somewhat inhibited 2,4-D metabolism in all biotypes (data not shown).

**Fig. 5.**
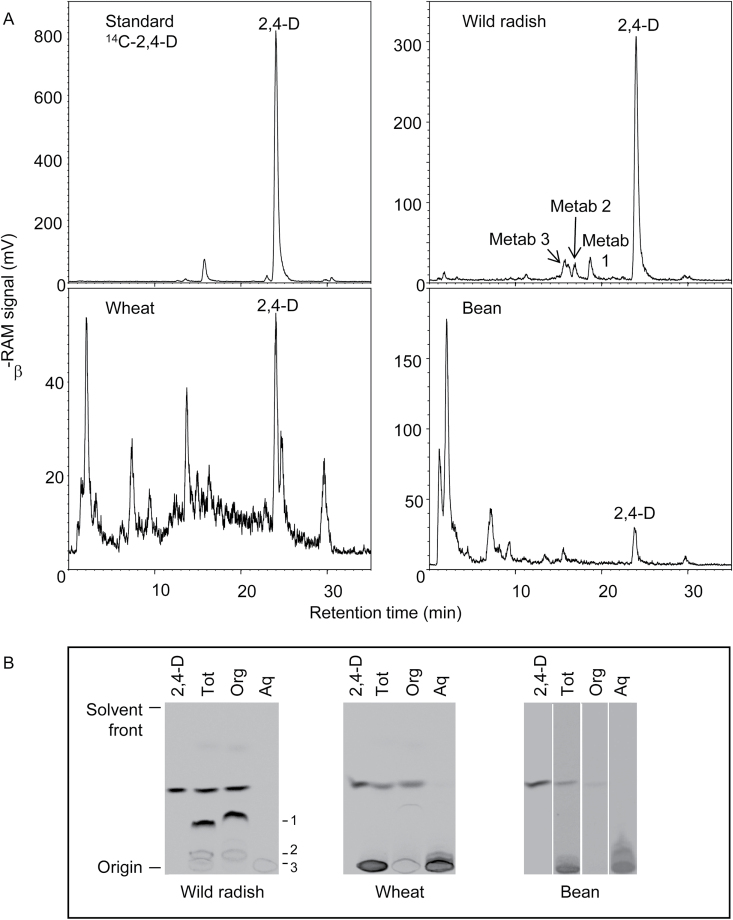
Comparison of [^14^C]2,4-D metabolism in wild radish (pooled biotypes), wheat, and bean. (A) Representative HPLC chromatograms of extracts from [^14^C]2,4-D-treated plants. Radioactivity was detected in the column eluent with a β-RAM detector. (B) Liquid–liquid partitioning of 2,4-D metabolites. Acidified extracts of [^14^C]2,4-D-treated plants were partitioned against diethyl ether to obtain the organic (Org) phase and then against 1-butanol to obtain the aqueous (Aq) phase, which were run on TLC plates alongside the total extract (Tot). Wild radish metabolites 1, 2, and 3 are indicated in (A) and (B). Representative HPLC chromatograms and TLC plates from three independent experiments are shown, except that wild radish metabolite 1 was often not detectable on HPLC.

**Fig. 6.**
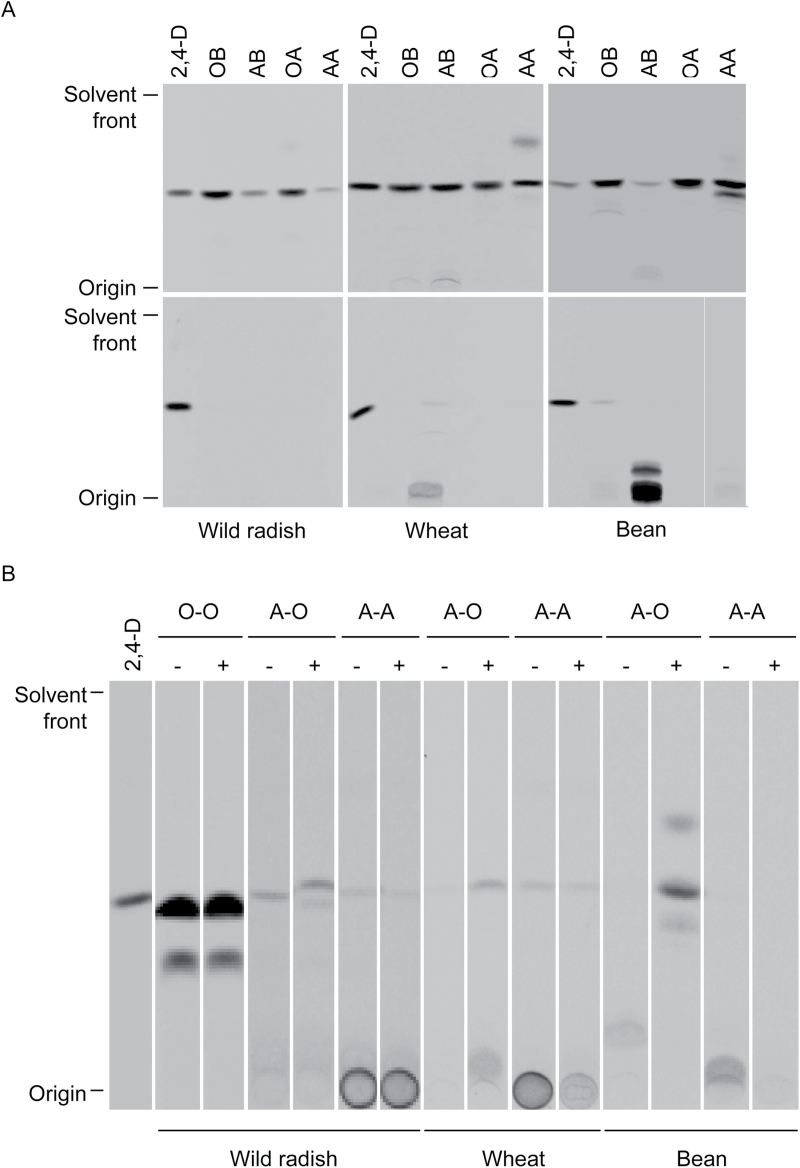
(A) Chemical hydrolysis of partitioned 2,4-D metabolites from wild radish (pooled biotypes), wheat, and bean. Organic fractions were hydrolysed with base (OB) or acid (OA), as were the aqueous fractions (AB and AA, respectively), and all were re-partitioned following hydrolysis (top panel, final organic phase; bottom panel, final aqueous phase). (B) Enzymatic hydrolysis of 2,4-D metabolites. Partitioned extracts from [^14^C]2,4-D-treated wild radish (pooled biotypes; organic and aqueous phases) and wheat and bean (aqueous phase only) plants were incubated in the absence (–) or presence (+) of β-glucosidase and then re-partitioned into organic and aqueous phases. Letters above lanes denote the phase of the original and post-hydrolysis fractions (i.e. O-O is the post-hydrolysis organic phase of the original organic fraction, A-O is the post-hydrolysis organic phase of the original aqueous fraction, and A-A is the post-hydrolysis aqueous phase of the original aqueous fraction). Representative TLC plates from three independent experiments are shown.

**Fig. 7.**
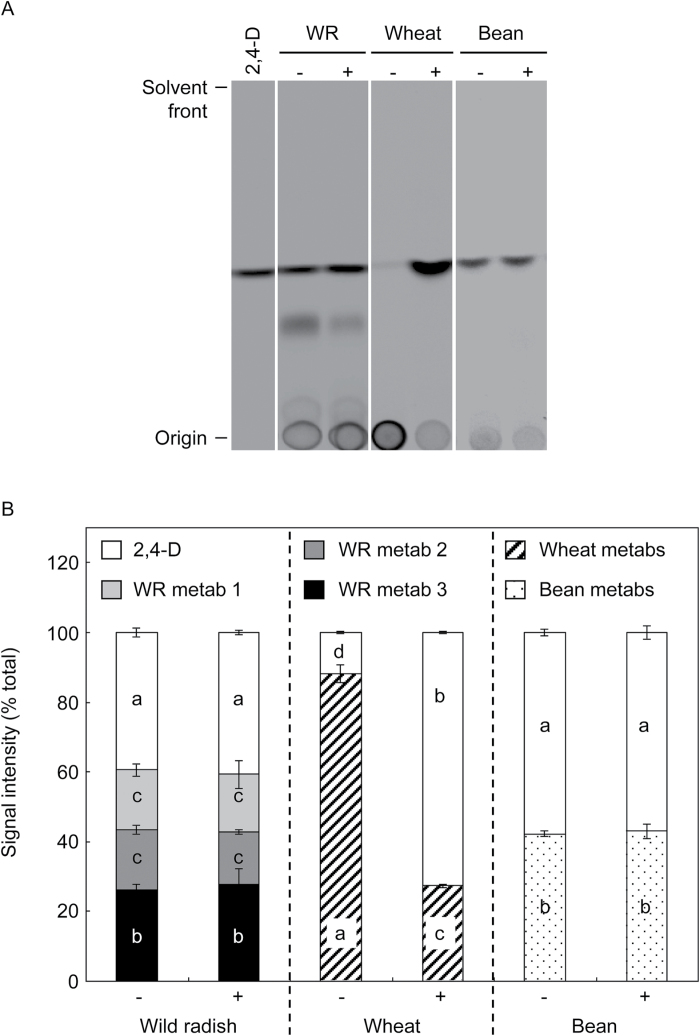
Effect of malathion on 2,4-D metabolism in wild radish (WR; pooled biotypes), wheat, and bean. Extracts of plants treated with (+) or without (–) malathion before the application of [^14^C]2,4-D were separated by TLC and the ^14^C signals on the TLC plates were quantified. Representative TLC plates are shown in (A); quantitative values in (B) are the mean ±SE of three independent experiments. Different letters within bars denote significant differences (*P*<0.05) between means (the data for wild radish, wheat, and bean were analysed separately).

Attempts to identify the wild radish 2,4-D metabolites using mass spectrometry have so far proved unsuccessful, so the chemical behaviour of the wild radish metabolites was compared with that of the well-characterized metabolites from wheat and bean (identified as predominantly carboxylic glucose esters and phenolic glycosides: Chkanikov *et al.*, 1976; Hamburg *et al.*, 2001). Representative HPLC and TLC separations of 2,4-D metabolites from plants harvested 96h after [^14^C]2,4-D application are shown in Fig. 5. Wheat and bean converted ~90% and 55% (see Fig. 7 for quantification), respectively, of absorbed [^14^C]2,4-D to polar metabolites which remained near the origin of TLC plates, had a retention time on HPLC of <15min, and were water soluble (Fig. 5). Wild radish produced a mixture of polar metabolites (which remained near the origin on TLC, had HPLC retention times of 16–17min, and were water soluble) and a prominent non-polar metabolite (TLC *R*_f_ 0.32; HPLC retention time 19min) which partitioned into the ether phase and appeared to be unstable under HPLC conditions, as it was not often visualized on HPLC chromatograms (Fig. 5). Mild acid and base hydrolysis of partitioned metabolites resulted in complete conversion of all the wild radish metabolites to a compound migrating identically to parent 2,4-D, whilst some of the wheat and almost all of the bean metabolites were resistant to mild base (Fig. 6A), indicative of phenolic glycosides (Chkanikov *et al.*, 1976). In contrast, the wild radish metabolites were essentially resistant to β-glucosidase while some of the wheat and all of the bean metabolites were hydrolysed by β-glucosidase treatment, converting to either parent 2,4-D or ether-soluble compounds that are likely to be hydroxylated aglycones (Feung *et al.*, 1971) (Fig. 6B). Pre-treatment of plants with the P450 inhibitor malathion caused a dramatic decrease in the capacity of wheat to metabolize 2,4-D, but had no effect on wild radish or bean (Fig. 7).

### Response of wild radish seedlings and leaves to auxins

The response of seedling root elongation to growth on agar containing auxins varied between biotype and auxin species. The R1 biotype was highly resistant to 2,4-D and MCPA, and moderately resistant to the other auxins, except IAA, when compared with the S biotype (Table 1). In contrast, the R2 biotype was moderately resistant only to 2,4-D and MCPA, and was in fact more sensitive to NAA than the S biotype (Table 1). Incubation of seedlings on agar containing [^14^C]2,4-D for 7 d showed that the label was present throughout the root system and also, more faintly, in the hypocotyl and cotyledons (Supplementary Fig. S3). Metabolism of 2,4-D in seedling roots, as detected by TLC, was negligible (Supplementary Fig. S3), with very faint signals from the polar metabolites and no detectable signal from the ether-soluble metabolite that is prominent in the leaf and shoot tissue (e.g. Fig. 4).

**Table 1.** Quantification of auxin resistance (root elongation response) in seedlings grown on auxin-containing medium

	**S**	**R1**		**R2**	
**Auxin**	**ED**_**50**_	**ED**_**50**_	**R:S**	**ED**_**50**_	**R:S**
2,4-D	0.009±0.002 c	5.3±0.8 a	604±157	0.2±0.04 b	27±7
MCPA	0.009±0.001 c	0.6±0.02 a	74±8	0.08±0.01 b	9±2
Mecoprop	0.04±0.01 b	0.3±0.02 a	6±1	0.05±0.01 b	1±0.2
Dicamba	0.3±0.1 b	3±0.6a	10±4	2±0.3 b	6±2
IAA	0.003±0.001 a	0.02±0.01 a	9±5	0.2±0.04 a	66±34
NAA	0.3±0.01 b	0.5±0.04 a	2±0.2	0.07±0.01 c	0.2±0.04

Root elongation data were fitted to dose–response curves and used to calculate the auxin concentration causing 50% inhibition of root elongation (ED_50_; μM ±SE) in the 2,4-D-susceptible (S) and -resistant (R1 and R2) biotypes.

The resistance index (R:S) is expressed as the ratio of ED_50_ values in the R and S biotypes.

Across rows, ED_50_ values with different letters are significantly different (*P*<0.05).

Seedlings incubated on agar containing 0.1 μM 2,4-D, or expanded leaves sprayed with the recommended rate of formulated 2,4-D amine, showed relatively minor changes in their tissue concentrations of ascorbate, glutathione, and H_2_O_2_ which did not appear to be correlated to the resistance status of the tissue (Supplementary Fig. S4). The reduction states of the ascorbate and glutathione pools did not change significantly in response to 2,4-D (Supplementary Fig. S4), although the E_GSSG/2GSH_ of the seedlings (average −160 mV) suggests that wild radish seedlings grown on agar may experience slight oxidative stress (Schafer and Buettner, 2001) regardless of the 2,4-D treatment.

## Discussion

### Reduced translocation of 2,4-D is the primary cause of 2,4-D resistance

The major 2,4-D resistance mechanism identified in the R1 and R2 wild radish biotypes is their inability to translocate 2,4-D away from its site of foliar application and into the growing points of the plant, as demonstrated by the whole-plant studies on [^14^C]2,4-D movement in the S and R biotypes (Fig. 1). Uptake of [^14^C]2,4-D applied to attached leaves of intact plants was very high in all biotypes, and there was no significant difference in the extent of influx of [^14^C]2,4-D into abraded leaf discs from S and R plants. Metabolism of 2,4-D also occurred to the same (relatively low) extent in the S and R biotypes (Fig. 5), which indicates that differential degradation or conjugation is not the reason for reduced translocation of 2,4-D in the resistant plants. Direct measurement of ^14^C in apoplastic and microsomal fractions demonstrated that [^14^C]2,4-D, or its metabolites, is unlikely to be sequestered in these compartments in any of the biotypes (Supplementary Table S2). The most likely candidate for storage of xenobiotics, the vacuole (Yuan *et al.*, 2007), could not be satisfactorily isolated from wild radish leaves, requiring an alternative strategy to investigate the potential vacuolar sequestration of 2,4-D.

As an indirect measure of intracellular (vacuolar) sequestration, leaf disc studies showed that after a rapid loss of two-thirds of the [^14^C]2,4-D that was taken up by the S and R leaf discs in the first 4h, there was a further slow decline in disc ^14^C until only 10% of the amount originally taken up by the discs remained in the tissue after 20h. This observation, coupled with the fact that the retention of the vacuolar stain Neutral Red was 2–3 times higher than that of ^14^C in the wild radish leaf discs between 4h and 20h, suggests that vacuolar sequestration of 2,4-D in wild radish leaves is negligible. Most of the ^14^C remaining in the wild radish leaf discs migrated identically to parent 2,4-D on TLC plates (Fig. 5C), further confirming that the residual ^14^C in the discs was unlikely to be stored in the vacuole. This is because vacuolar sequestration of herbicides usually (but not always) requires the herbicide to be conjugated to sugar or glutathione in order for the vacuolar transporters to recognize their substrate (e.g. Yuan *et al.*, 2007). Additionally, the chemiosmotic hypothesis states that weakly acidic molecules such as unmodified IAA (p*K*_a_ 4.8) and 2,4-D (p*K*_a_ 2.7) will be partially protonated (i.e. uncharged) in an acidic environment such as in the plant apoplast or vacuole (pH ~5.5) and thus able to diffuse through a lipid membrane; upon reaching the cytosol (pH ~7), the molecules become fully deprotonated and consequently trapped in the cytosol (e.g. Carrier *et al.*, 2008). Therefore, a proportion of any parent 2,4-D actively transported across the tonoplast membrane from the neutral cytosol to the acidic vacuole would be expected to diffuse back into the cytosol.

### Inhibition of phloem loading is potentially responsible for reduced translocation of 2,4-D

Having ruled out differential leaf 2,4-D uptake, metabolism, or sequestration as contributing to the observed differences in 2,4-D translocation between the S and R biotypes, the focus turns to the mechanics of 2,4-D transferral from the mesophyll cells into the phloem. A degree of qualitative support for the phloem-loading hypothesis is provided by the ^14^C signal in [^14^C]2,4-D-treated plants: the signal clearly shows up in the major and minor veins of the treated leaf of the S biotype, whereas the treated leaf of the R biotypes, and of S plants exposed to transport inhibitors, is almost a ‘negative’ of this picture, with the signal being present in the mesophyll but excluded from the veins (see treated leaf enlargements in Fig. 1H–N, particularly those from Fig. 1H versus 1J and M). In plants, the ABCB-type auxin efflux transporters are involved both in cell to cell polar auxin transport and in facilitating long-distance transport of auxin through the phloem by preventing its influx into the cells adjacent to the vascular bundle (Reemmer and Murphy, 2014). A number of protein modelling and cell-based auxin transport studies have indicated that 2,4-D can bind to ABCB1 (Bailly *et al.*, 2012), ABCB4 (Kubeš *et al.*, 2012), and ABCB19 (Yang and Murphy, 2009), although there is little direct evidence that 2,4-D is moved across the plasma membrane by these transporters. The fact that known inhibitors of ABCB-type transporters, verapamil and valspodar, inhibited [^14^C]2,4-D translocation in the current study (Figs 1, 2) provides additional indirect evidence that an ABCB transporter may facilitate long-distance movement of 2,4-D in wild radish. Efficient ABCB-type auxin efflux transporter activity is dependent upon the immunophilin-like protein TWD1, which plays an essential role in trafficking ABCB proteins to the plasma membrane (Wu *et al.*, 2010). The auxin efflux inhibitor NPA binds to both TWD1 and the ABCB-type auxin transporters, disrupting their function (Kim *et al.*, 2010).

Based on the observed inhibitory effect of NPA on [^14^C]2,4-D translocation in the S wild radish biotype (Fig. 2A), it is tempting to speculate that reduced 2,4-D translocation in the R biotypes tested in this study is due to a change in sequence, expression or localization of one of the NPA-binding proteins involved in long-distance auxin transport. It should also be noted that TIBA, an auxin efflux inhibitor that does not interfere with the interaction between ABCB transporters and TWD1 (Bailly *et al.*, 2008), also efficiently inhibited [^14^C]2,4-D translocation in S plants. TIBA is believed to act by disrupting intracellular movement of the PIN transporters (Zhu and Geisler, 2015), suggesting either that PIN proteins (some of which have been shown to bind 2,4-D; Yang and Murphy, 2009) also contribute to long-distance transport of 2,4-D, or that disruption of PIN trafficking by TIBA upsets ABCB-mediated 2,4-D transport. This is a possibility because a direct interaction of PIN1 with ABCB1 and ABCB19, possibly regulated by TWD1, has been demonstrated in certain tissues of Arabidopsis seedlings (Blakeslee *et al.*, 2007). Taken all together, the findings from this and previous studies strengthen the possibility that an ABCB-type auxin transporter is involved in phloem loading of auxinic herbicides as well as of natural auxin, and that this is the point at which 2,4-D resistance has developed in wild radish biotypes R1 and R2. The unexpected stimulation of [^14^C]2,4-D translocation in verapamil-treated R plants requires further investigation. Until the resistance-conferring protein is unequivocally identified, it can only be speculated that perhaps binding of verapamil to a mutated or poorly localized ABCB transporter partially restores its function, although this seems unlikely.

### Cross-resistance to other synthetic auxins

The wild radish R1 and R2 biotypes showed much less inhibition of root elongation than the S biotype when grown on agar containing 2,4-D and MCPA (and, in the case of R1, also mecoprop, dicamba, and NAA) (Table 1). The observed lack of [^14^C]2,4-D metabolism by the root tissue means that herbicide detoxification in the R biotypes is unlikely to explain the difference in root elongation response to 2,4-D, at least. The fact that seedlings grown on [^14^C]2,4-D showed a ^14^C signal distributed throughout the root tissue indicates that there were no differences in bulk 2,4-D translocation or accumulation as was observed in the shoots, but, as individual cells cannot be resolved by phosphorimaging, it is still possible that 2,4-D was excluded from the root apical meristem in the R biotypes. The difference between root elongation in the S and R biotypes was also not due to enhanced antioxidant capacity in the latter. Differences in response to different auxin classes can be due to mutations in specific auxin receptors or proteins associated with the signal transduction pathway, with the classical example being the picolinate-resistant Arabidopsis mutant, which has an alteration in the AFB5 auxin receptor (Walsh *et al.*, 2006). The ABCB transporters can also show auxin selectivity, with ABCB21 (Kamimoto *et al.*, 2012) and particularly ABCB19 (Bouchard *et al.*, 2006) showing higher export activity with IAA than with NAA as a substrate, and ABCB4 (a root-localized auxin transporter) having a higher affinity for 2,4-D than does ABCB19 (Kubeš *et al.*, 2012). The reason for differential auxin resistance at the root elongation level in R1 and R2 currently remains speculative, but the observed low (R1) or lack of (R2) resistance to NAA (Table 1) could be construed as involving ABCB19 or ABCB21 in resistance to 2,4-D and MCPA. Alternatively, the root-localized ABCB4 could play a role, as root hair growth in Arabidopsis *abcb4* mutants is resistant to moderate concentrations of 2,4-D (Kubeš *et al.*, 2012). Preliminary genetic analysis of 2,4-D resistance in R1, using the survival of foliar-sprayed F_1_ and F_2_ crosses between the S and (unselected) R1 biotypes as a measure of resistance, suggests that resistance in R1 shoots is likely to be conferred by a single, semi-dominant gene (R. Busi, personal communication), but the number and nature (i.e. transporters, receptors, signal transduction complexes) of genes conferring auxin resistance at the root elongation level is currently unknown in both R1 and R2.

### Fate of 2,4-D in wild radish

The similarity in 2,4-D metabolism between the S and R wild radish biotypes, and the high proportion of parent 2,4-D and ether-soluble metabolite (these are usually herbicidally active; Feung *et al.*, 1977) recovered from these plants, confirms that enhanced metabolic 2,4-D detoxification does not contribute to resistance in wild radish biotypes R1 and R2. A comparison of the chemical properties of 2,4-D metabolites in wild radish, wheat, and bean suggests that in contrast to the latter two species, wild radish does not hydroxylate 2,4-D to an appreciable degree as its metabolites were not resistant to mild base hydrolysis, which is a characteristic of phenolic glycosides of 2,4-D (Chkanikov *et al.*, 1976). As a sideline, the dramatically different effect of malathion on 2,4-D metabolism in wheat and bean provides indirect evidence that the so far uncharacterized 2,4-D-hydroxylating activity in plants is represented by a cytochrome P450 mono-oxygenase in wheat and a non-P450 (or a P450 uninhibited by malathion) in bean.

Another interesting feature of the wild radish 2,4-D metabolites was that both the polar and non-polar compounds were resistant to β-glucosidase digestion, suggesting that glucose is not conjugated to 2,4-D in this species. The ether-soluble wild radish metabolite could be an amino acid conjugate, but these are usually resistant to acid hydrolysis (Chkanikov *et al.*, 1976), which was not the case in this study. An *in vitro* glutathione *S*-transferase assay using wild radish leaf extract with [^14^C]2,4-D as a substrate gave no reaction products (data not shown), suggesting that glutathione is not conjugated to 2,4-D in wild radish as is potentially the case in potato (Peixoto *et al.*, 2008) and dog (van Ravenzwaay *et al.*, 2003). Characterization of the 2,4-D metabolites produced by wild radish, initiated in this study but requiring more work, is necessary so that any future populations evolving metabolic 2,4-D resistance and displaying ‘new’ metabolites can be rapidly identified.

In summary, the reduced-translocation mechanism of field-evolved 2,4-D resistance in the two wild radish biotypes studied here appears to be due to a lack of transport of parent 2,4-D into and through the phloem, rather than to differential uptake, metabolism, and/or sequestration of the applied 2,4-D. The proposed involvement of an altered ABCB auxin efflux transporter in mediating 2,4-D resistance in these biotypes needs to be directly demonstrated, and this is the focus of ongoing investigations. In addition to this major resistance mechanism, it appears that foliar spraying of 2,4-D onto wild radish populations in the field has resulted in the selection of a systemic change in auxin transport, perception, or signalling which is expressed as a minor resistance mechanism in the roots.

## Supplementary data

Supplementary data are available at *JXB* online.

Table S1. Comparison of direct and indirect measurement of influx of [^14^C]2,4-D into wild radish leaf discs.

Table S2. Marker enzyme activity and ^14^C in apoplast and microsome fractions from [^14^C]2,4-D-treated leaves from the S, R1. and R2 biotypes.

Figure S1. Appearance of 2,4-D-treated and -untreated seedlings of the S, R1, and R2 biotypes.

Figure S2. Phosphorimages of [^14^C]2,4-D translocation in plants of the R1 and R2 biotypes treated with transport inhibitors.

Figure S3. [^14^C]2,4-D metabolism in wild radish leaf discs and roots, and distribution of ^14^C in seedlings grown on [^14^C]2,4-D-containing agar.

Figure S4. Ascorbate, glutathione, and H_2_O_2_ concentrations in whole seedlings and expanded leaves of 2,4-D-treated S, R1, and R2 biotypes.

Supplementary Data
