# Supplementary material for: 2,4-D resistance in wild radish: reduced herbicide translocation via inhibition of cellular transport
Source: J Exp Bot. 2016 Mar 19;67(11):3223–35. doi: 10.1093/jxb/erw120 (PMC4892717; doi:10.1093/jxb/erw120)
Supplement: Supplementary Data [file supp_67_11_3223__index.html]

2,4-D resistance in wild radish: reduced herbicide translocation via inhibition of cellular transport — 2,4-D resistance in wild radish: reduced herbicide translocation via inhibition of cellular transport — Supplementary Data 

# 2,4-D resistance in wild radish: reduced herbicide translocation via inhibition of cellular transport

## Supplementary Data

Data files

- supplementary\_tables\_S1\_S2\_figures\_S1\_S4.pdf - Supplementary Data
