# Supplementary material for: 2,4-D resistance in wild radish: reduced herbicide translocation via inhibition of cellular transport
Source: J Exp Bot. 2016 Mar 19;67(11):3223–35. doi: 10.1093/jxb/erw120 (PMC4892717; doi:10.1093/jxb/erw120)
Supplement: Supplementary Data [file supp_erw120_supplementary_tables_S1_S2_figures_S1_S4.pdf]

Journal of Experimental Botany

2,4-D resistance in wild radish: reduced herbicide translocation via inhibition of phloem loading

Authors: D Goggin, G Cawthray & S Powles.

Supplementary Table S1. *Comparison of direct and indirect measurement of influx of ^{14}C-2,4-D (% applied) into wild radish leaf discs.*

Biotype	Direct measurement	Indirect measurement
S	39±1	43±2
R1	46±2	48±1
R2	46±4	45±2

The amount of ^{14}C-2,4-D taken up by leaf discs from each of the susceptible (S) and resistant (R1 and R2) biotypes was measured directly by harvesting and extracting discs immediately after the 60 min uptake phase, or indirectly by summing the ^{14}C in the efflux buffer and the leaf discs at the end of the 120 min efflux phase (see Materials and Methods). Influx of ^{14}C-2,4-D is expressed as a % of ^{14}C-2,4-D applied; analysis of variance indicated no significant differences (P = 0.2) between measurement techniques or between biotypes.

Supplementary Table S2. *Marker enzyme activity and ^{14}C in apoplast and microsome fractions from ^{14}C-2,4-D-treated leaves from the S, R1 and R2 biotypes.*

**Apoplast**		**S**	**R1**	**R2**
Malate dehydrogenase	Apoplast	2.6±1.1[a]	2.5±0.9[a]	2.3±0.4[a]
(µmol min^{-1} g^{-1} fwt)	Symplast	34±11[a]	32±7[a]	31±3[a]
^{14}C	Apoplast	4.8±0.7[a]	7.8±1.6[a]	7.7±1.2[a]
(% of total recovered from tissue)	Symplast	64±1[a]	61±1[a]	61±1[a]
**Microsomes**		**S**	**R1**	**R2**
Malate dehydrogenase	Microsome	3.0±0.7[a]	2.3±0.2[a]	2.3±0.1[a]
(µmol min^{-1} mg^{-1} protein)	Soluble[a]	4.1±0.4[a]	4.5±0.3[a]	4.5±0.1[a]
NADPH-cytochrome c reductase	Microsome	20±2[a]	21±6[a]	20±1[a]
(nmol min^{-1} mg^{-1} protein)	Soluble	2±2[a]	3±1[a]	5±2[a]
^{14}C	Microsome	2.6±0.1[b]	3.2±0.0[a]	3.1±0.2[a]
(% of total recovered from tissue)	Soluble	80±2[a]	78±2[a]	74±1[a]

Values are means ± SE (n = 3, with three leaves from different individuals per replicate). Across rows, values with different letters are significantly different ($P < 0.05$).

[a]'Soluble' refers to the combined contents of the cytosol, mitochondria, plastids and vacuoles.

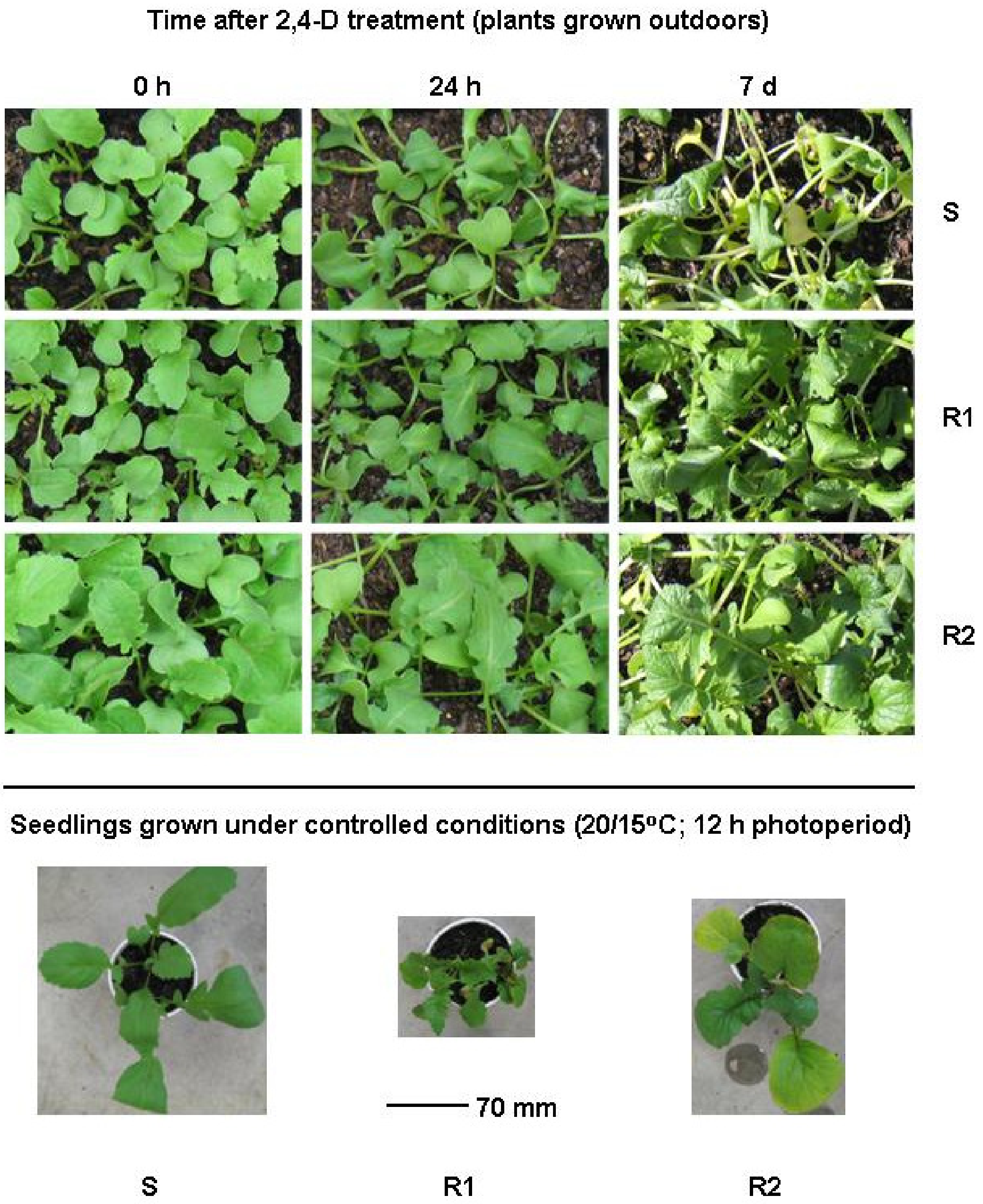

Supplementary Fig. S1. Appearance of seedlings from the 2,4-D-susceptible (S) and –resistant (R1 and R2) biotypes. The top panel shows seedlings grown outdoors in trays, before and after (24 h and 7 d) spraying with the recommended rate (500 g ha^{-1}) of formulated 2,4-D amine. The bottom panel shows untreated seedlings grown under controlled conditions in polystyrene cups at 21 d after sowing. Cups contain one (R2) or two (S and R1) seedlings.

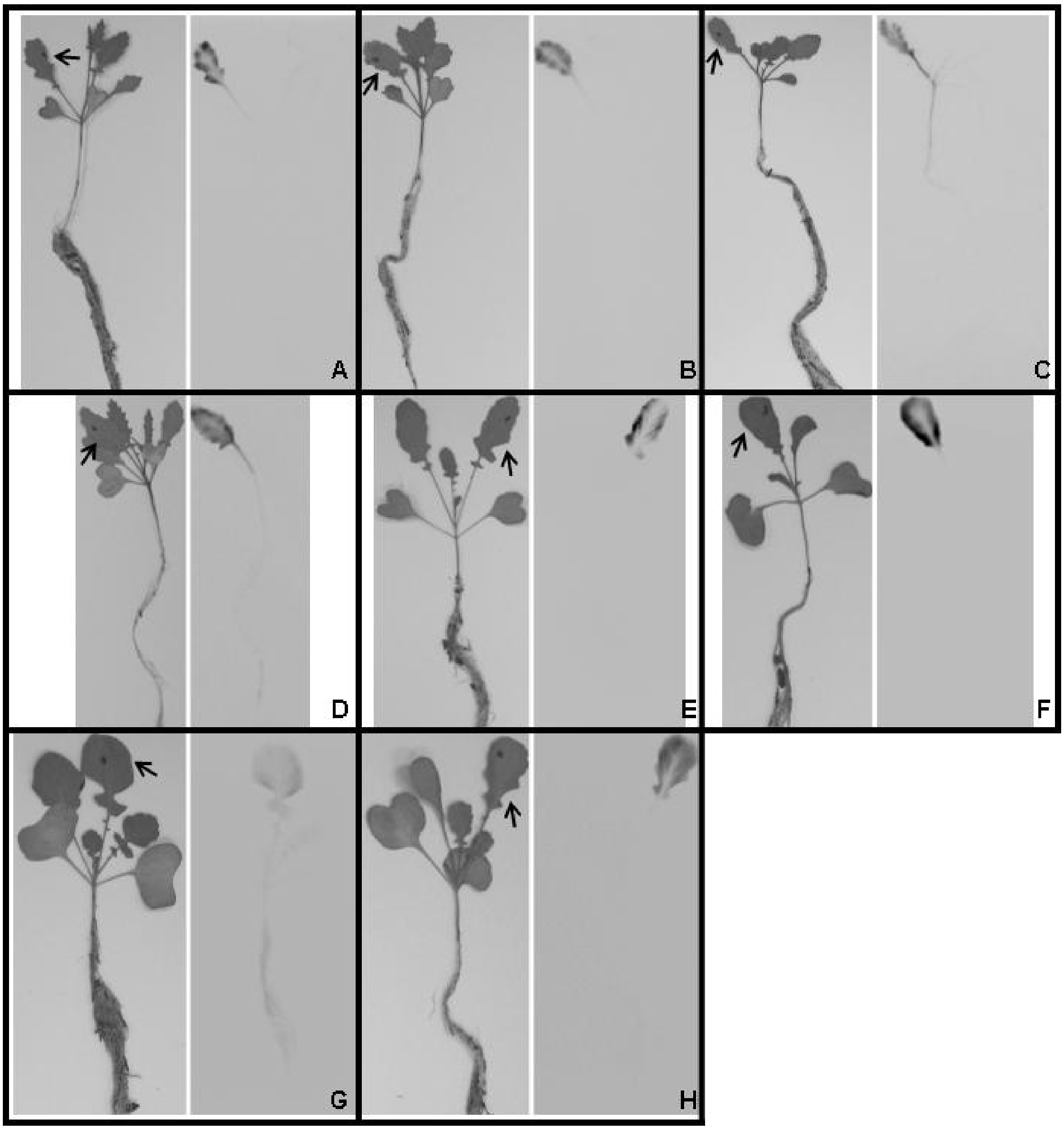

Supplementary Fig. S2. Photographs (left panels) and phosphorimages (right panels) of plants from the R1 (A – D) and R2 (E – F) biotypes treated with 10 µM NPA (A, E), TIBA (B, F), verapamil (C, G) or valspodar (D, H), applied via the hydroponic nutrient solution 8 h before ^{14}C-2,4-D was applied to a single leaf (marked with an arrow). Plants were harvested 24 h after ^{14}C-2,4-D was applied. Untreated controls are shown in Fig. 1B and C.

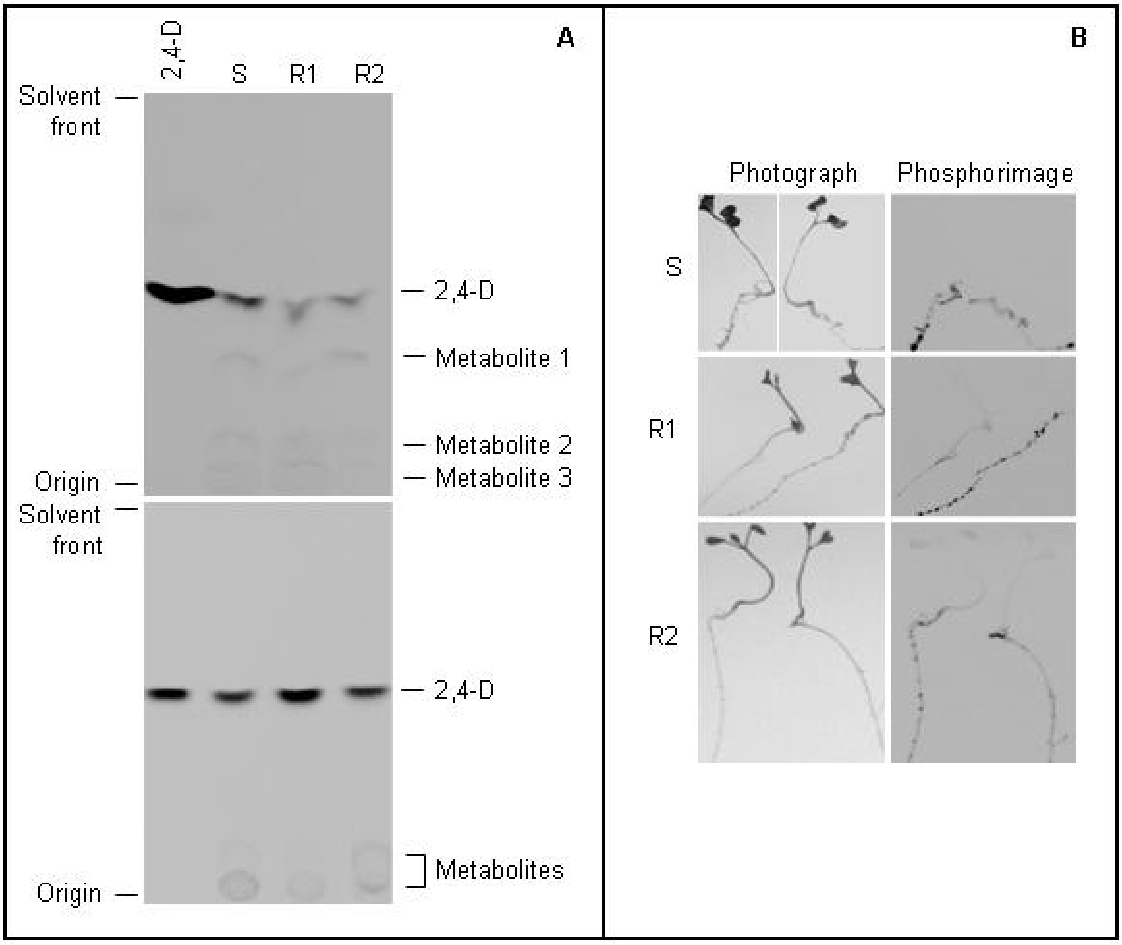

Supplementary Fig. S3. (A) Top panel: Composition of ^{14}C-labelled compounds remaining in ^{14}C-2,4-D-loaded leaf discs, after 16 h incubation in ^{14}C-free efflux buffer. TLC plates were loaded with 70 Bq per lane of leaf disc extracts from susceptible (S) and resistant (R1 and R2) plants and exposed to the imaging plate for 90 h. A representative image from two independent experiments is shown. Bottom panel: TLC of root extracts from seedlings (12 per biotype) grown hydroponically in ^{14}C-2,4-D-containing medium for 96 h, with 670 Bq loaded per lane. (B) Distribution of ^{14}C in seedlings grown on agar containing ^{14}C-2,4-D for 7 d, visualised by phosphorimaging of the seedlings.

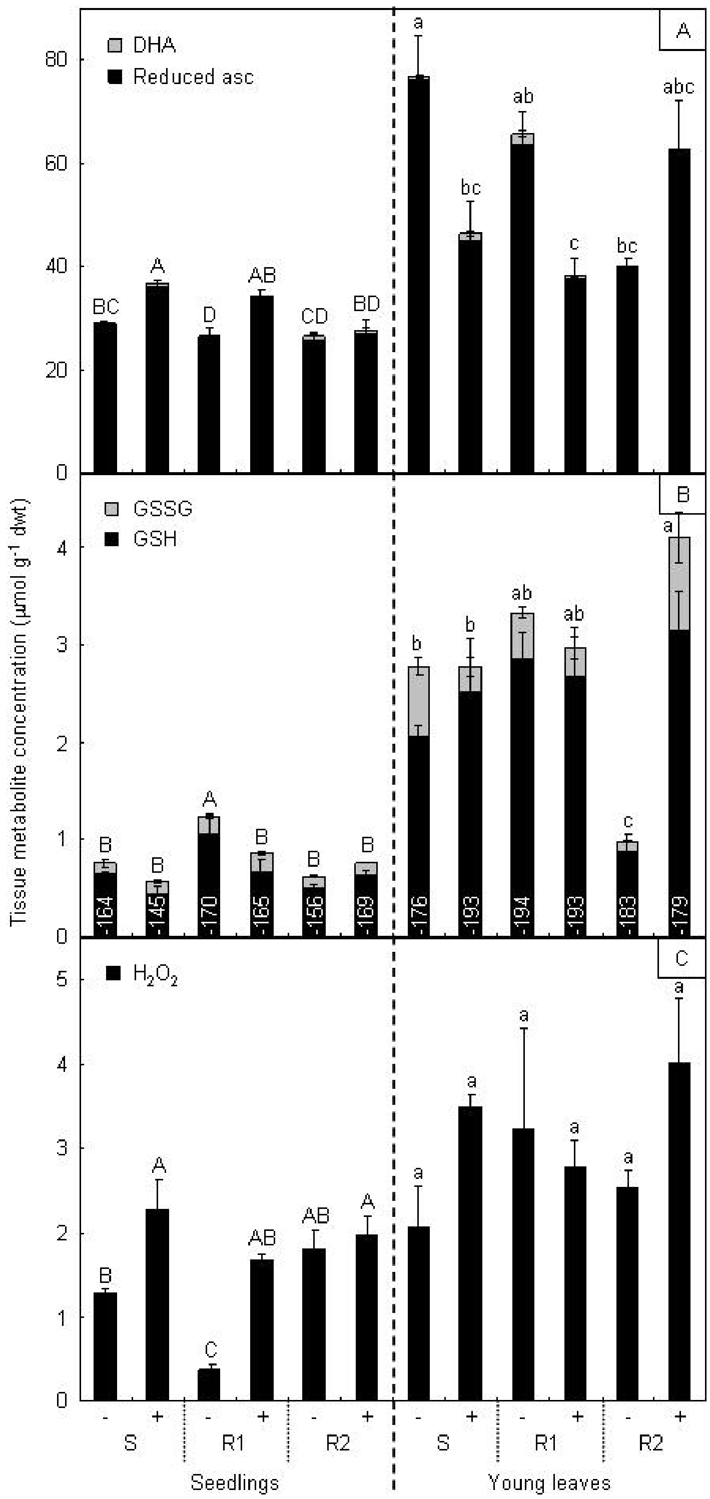

Supplementary Fig. S4. Response of tissue antioxidants and H_2O_2 to 2,4-D treatment. Seedlings of the susceptible (S) and resistant (R1 and R2) biotypes were incubated on

agar containing 0.1 µM 2,4-D for 3 d, whilst young, expanded leaves on soil-grown S, R1 and R2 plants at the 4- to 5-leaf stage were sprayed with 500 g ha^{-1} formulated 2,4-D amine and harvested after 24 h. Concentrations of (A) reduced ascorbate (asc) and dehydroascorbate (DHA), (B) reduced (GSH) and oxidised (GSSG) glutathione and (C) H_2O_2 were measured in tissue extracts. Values are means ± SE (for the seedling study n = 3, with 5 seedlings per replicate; for the leaf study, n = 6 replicates of two or three leaves from different individuals for ascorbate and glutathione, and n = 3 replicates for H_2O_2). White numbers inside the bars in (B) are the $E_{GSSG/2GSH}$ (mV) of the glutathione pool. Significant differences between total pool sizes (*P*<0.05) are denoted by different letters above the bars; the seedlings and leaves were analysed separately, as indicated by the use of upper- and lower-case letters, respectively.
